# Dual Remission in Patients with Moderate-to-Severe Asthma and CRSwNP Treated with Dupilumab: A 24-Month Real-World Study

**DOI:** 10.3390/jcm15072787

**Published:** 2026-04-07

**Authors:** Francesco Menzella, Alvise Berti, Walter Cestaro, Annamaria Bosi, Sara Munari, Francesco Gialdini, Tatiana Scandiuzzi Piovesan, Marcello Cottini, Carlo Lombardi, Lorenzo Corsi, Eugenio De Corso

**Affiliations:** 1 Pulmonology Unit, S. Valentino Hospital, AULSS2 Marca Trevigiana, 31044 Montebelluna, TV, Italy; 2 Multidisciplinary Pulmonology and Otolaryngology Unit, S. Valentino Hospital, AULSS2 Marca Trevigiana, 31044 Montebelluna, TV, Italy; 3 CISMED, Department of CIBIO, University of Trento, Unit of Rheumatology, S. Chiara Hospital, ASUIT, 38123 Trento, Italy; 4 Otolaryngology Unit, S. Valentino Hospital, AULSS2 Marca Trevigiana, 31044 Montebelluna, TV, Italy; 5 Allergy and Pneumology Outpatient Clinic, 24100 Bergamo, Italy; 6 Departmental Unit of Allergology, Immunology and Pulmonary Diseases, Fondazione Poliambulanza, 25124 Brescia, Italy; 7 Otorhinolaryngology-Head and Neck Surgery, University of Perugia, 06132 Perugia, Italy

**Keywords:** asthma, chronic rhinosinusitis with nasal polyps, dupilumab, dual remission, real-world evidence, type 2 inflammation

## Abstract

**Background:** “Remission” is a primary therapeutic goal in severe asthma and chronic rhinosinusitis with nasal polyps (CRSwNP), though definitions vary regarding olfactory function. We evaluated “Dual Remission” kinetics in patients treated with dupilumab over 24 months. **Methods:** This single-center retrospective study analyzed 28 patients with comorbid severe asthma and CRSwNP. Dual Remission was defined as simultaneous asthma remission (ACT ≥ 20, no exacerbations, no OCS and stable lung function) and CRSwNP remission (SNOT-22 < 40, NPS ≤ 1). We additionally analyzed “Complete Recovery” by applying a stricter composite definition requiring the restoration of normosmia (Sniffin’ Sticks score ≥ 12). **Results:** At baseline, patients exhibited uncontrolled disease (median ACT 19, NPS 6). Treatment led to rapid asthma remission (85.7% at 12 months, 100% at 24 months). CRSwNP remission was slower but progressive, rising from 57% at 12 months to 88% at 24 months, demonstrating a significant “catch-up” phenomenon. Consequently, Dual Remission rates increased from 54% to 88% by month 24. When applying the stricter “Complete Recovery” criteria requiring normosmia, only 32% met the goal. **Conclusion:** Dupilumab is highly effective, enabling 88% of patients to achieve Dual Remission after 24 months. However, full olfactory restitution is distinct from structural polyp regression and harder to achieve, likely due to persistent neuroepithelial damage.

## 1. Introduction

Asthma and Chronic Rhinosinusitis with Nasal Polyps (CRSwNP) are frequently comorbid conditions, linked by a shared pathogenic mechanism known as Type 2 (T2) inflammation [1]. This inflammatory pathway is driven by key cytokines, particularly interleukin-4 (IL-4), IL-13 and IL-5, which recruit eosinophils and drive tissue remodeling in both the upper and lower airways [1,2]. Dupilumab, a fully human monoclonal antibody, targets the IL-4 receptor alpha subunit (IL-4/13 R α), effectively blocking the signaling of both IL-4 and IL-13 [3].

In long-term clinical studies of dupilumab, approximately 11% of patients with CRSwNP and comorbid asthma achieved clinical remission of nasal polyposis after 12 months of treatment, rising to 31% at 24 months based on comprehensive remission definitions. In Type 2 asthma clinical trials (QUEST and TRAVERSE), around 37% of patients on dupilumab met asthma remission criteria at 12 months and ~43% at 24 months, significantly higher than multicomponent clinical placebo, demonstrating meaningful dual disease control with this biologic therapy [4,5].

While these clinical trials have demonstrated its efficacy in reducing exacerbations and polyp size individually, there is a scarcity of real-world data assessing simultaneous clinical remission, i.e., dual remission. This concept—reaching strict treat-to-target goals in both nose and lungs concurrently—represents a higher standard of care than merely controlling symptoms.

This study aims to fill this gap by evaluating the effectiveness of dupilumab on asthma and nasal polyps over a 24-month period in a retrospective monocentric cohort of comorbid severe Type 2 asthma and CRSwNP, specifically tracking the kinetics of this dual remission.

## 2. Materials and Methods

### 2.1. Study Design and Population

This single-center, observational, retrospective study was conducted at the Multidisciplinary Pulmonology and Otolaryngology Unit of the S. Valentino Hospital in Montebelluna (TV) in a real-life setting involving consecutive patients (>18 years old) suffering from moderate-to-severe asthma and CRSwNP. Given the retrospective, real-life nature of this study, the sample size was determined by the total number of patients meeting the inclusion criteria within the study period. All patients with comorbid severe asthma and CRSwNP who initiated treatment with dupilumab at our Multidisciplinary Unit during the study period and met the pre-defined inclusion criteria were included in the analysis. No patients were excluded based on treatment response or clinical outcomes. All subjects were treated with dupilumab (initial dose of 600 mg followed by 300 mg every 2 weeks) as an add-on therapy. Inclusion criteria were a diagnosis of severe asthma according to GINA guidelines and concomitant CRSwNP eligible for biological treatment [6]. Data were collected at baseline (T0) and after 6 (T6), 12 (T12), and 24 (T24) months of treatment. Data analysis for this study commenced in September 2025.

During the preparation of this manuscript, the authors used Gemini 3 Pro for language editing and clarity. The authors have reviewed and edited the output and take full responsibility for the content of this publication.

### 2.2. Clinical and Functional Assessment

At each visit, patients underwent a multidisciplinary assessment. Asthma control was evaluated using the Asthma Control Test (ACT) [7], stable or improved forced expiratory volume in 1 s (FEV_1_), number of exacerbations, and use of oral corticosteroids (OCSs). Lung function was assessed according to ERS/ATS technical standards [8]. Nasal outcomes were evaluated using the Sino-Nasal Outcome Test-22 (SNOT-22) for Quality of Life [9] and the Nasal Polyp Score (NPS) via nasal endoscopy (scored 0–4 per nostril, total 0–8) [10,11]. Olfactory function was assessed using the Sniffin’ Sticks Identification test (SS-I), where a score < 12 indicates hyposmia/anosmia and ≥12 indicates normosmia. “Complete Recovery” added a requirement for normosmia (SS-I ≥ 12). Type 2 inflammation biomarkers (blood eosinophils, total IgE, and FeNO) were also recorded.

### 2.3. Definition of Clinical Outcomes

The primary outcome was the achievement of “Dual Remission.“ Asthma Remission was defined according to the Severe Asthma Network Italy (SANI) criteria as the simultaneous presence of: (1) no OCS use, (2) no exacerbations, (3) ACT score ≥ 20, and (4) stable pulmonary function (FEV_1_) [12]. CRSwNP Remission was defined according to EPOS/EUFOREA guidelines as: (1) an NPS ≤ 1 and (2) a SNOT-22 score < 40 [13]. Dual remission was defined as meeting the criteria for both asthma remission and CRSwNP remission simultaneously. To evaluate the full restoration of sensory function, we additionally calculated a stricter composite endpoint termed “Complete Recovery” (Dual Remission + normosmia). This stricter definition required patients to meet all criteria for Dual Remission and additionally achieve normosmia, defined as a SS-I test score of ≥12 [14].

### 2.4. Statistical Analysis

Continuous variables are reported as medians and interquartile ranges (IQRs). Categorical variables are reported as counts and percentages. Comparisons between time points were performed using the Wilcoxon signed-rank test for paired data. In longitudinal figures, data are presented as medians with 95% Confidence Intervals (95% CIs) to ensure consistent reporting of non-parametric distributions. A *p*-value < 0.05 was considered statistically significant.

## 3. Results

### 3.1. Baseline Patient Features

**Figure 1:**
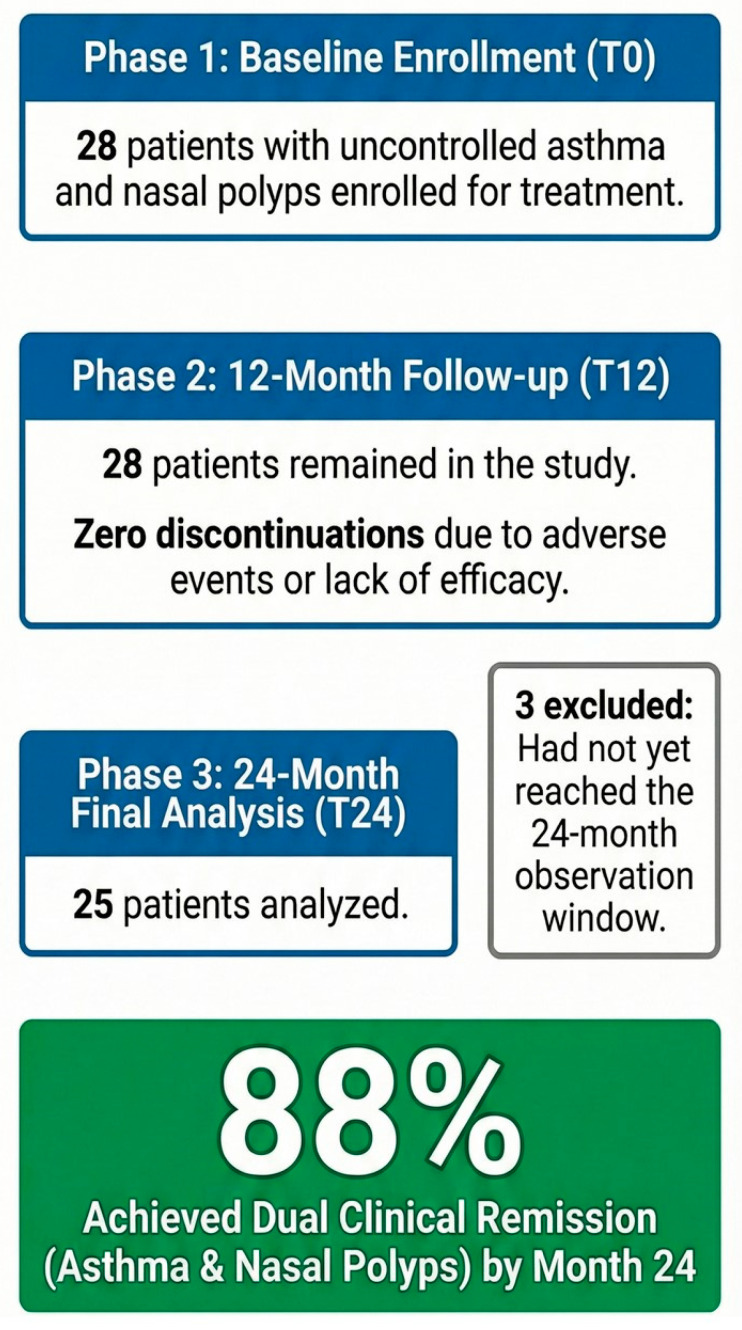
Study Flowchart (CONSORT-style). The diagram illustrates the selection and follow-up process of the study population. Initially, 28 consecutive patients with comorbid severe asthma and CRSwNP were assessed and initiated treatment with dupilumab. All 28 patients completed the 12-month clinical and functional assessment. At the 24-month follow-up, 25 patients were included in the final analysis. Three patients (n = 3) were excluded from the 24-month timepoint solely because they had not yet reached the required observation window at the time of data lock. Notably, no patients discontinued the study due to adverse events or lack of efficacy, ensuring a 0% clinical attrition rate during the 24-month period.

Twenty-eight patients (mean age 54.2 ± 12.8, 43% females) were enrolled and included in the baseline and 12-month analyses. To minimize selection bias, we employed a consecutive enrollment strategy of all eligible patients as illustrated in the CONSORT-style flow diagram (Figure 1). The high retention rate is demonstrated by the absence of any treatment discontinuations due to safety or efficacy concerns over the entire 24-month study period. The slight reduction in sample size at the final timepoint (*n* = 25) was due to temporal administrative reasons (data lock) rather than clinical drop-outs. No discontinuations due to adverse events or lack of efficacy were recorded during the study period. The median history of asthma was almost 12 years, and the median history of sinusitis was approximately 12.5 years; furthermore, 71% had moderate asthma where the primary biologic indication was severe CRSwNP. Analysis of the baseline asthma severity (Table 1) revealed that 8 patients (29%) were affected by severe asthma, characterized by high-dose Inhaled Corticosteroids (ICSs) and Long-Acting Beta-Agonists (LABAs) often combined with Long-Acting Muscarinic Antagonists (LAMAs) or OCS dependence. The remaining 20 patients (71%) presented with moderate asthma, where the primary indication for biologic therapy was the uncontrolled severe CRSwNP burden. All patients had uncontrolled disease at inclusion, with a mean SNOT-22 score of 65.7 and a median NPS of 6.0. Asthma was poorly controlled (median ACT 19) and patients were reliant on OCS (median 1.40 mg/day).

### 3.2. Clinical Evolution

**Figure 2:**
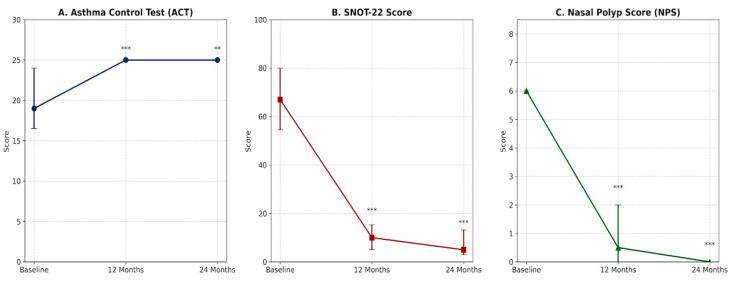
Longitudinal trajectories of asthma and sinonasal disease activity over 24 months. Graphs illustrate the evolution of clinical parameters: (**A**) ACT, (**B**) SNOT-22, and (**C**) NPS. Data are presented as medians, and error bars represent the 95% Confidence Intervals. Significant improvements (*p* < 0.05) were observed for all parameters compared to baseline using the Wilcoxon signed-rank test. Data points represent medians, and error bars indicate the 95% Confidence Intervals. Statistical significance compared to baseline was determined using the Wilcoxon signed-rank test and is denoted as follows: ** *p* < 0.01; *** *p* < 0.001. Abbreviations: ACT: Asthma Control Test; SNOT-22: Sino-Nasal Outcome Test-22; NPS: Nasal Polyp Score.

To ensure statistical consistency, Figure 2 presents all longitudinal clinical scores (ACT, SNOT-22, NPS) as medians with 95% Confidence Intervals (CIs). Treatment resulted in dramatic improvements across all clinical parameters over the 24-month period (Table 2, Figure 2). Asthma control improved strongly at 12 months (median ACT increasing from 19 to 25) and remained stable at 24 months. Sino-nasal symptoms ameliorated, with median SNOT-22 scores dropping from 67 at baseline to 10 at 12 months, and 5 at 24 months. Nasal polyps significantly reduced from a median NPS of 6.0 to 0.5 at 12 months and 0.0 at 24 months.

Assessment of olfactory function using the SS-I test revealed a marked recovery in olfactory capacity among treated patients. The median olfactory identification score increased significantly from a baseline value of 3.0 (IQR 3.0–3.0) to 7.0 (IQR 5.25–9.75) after 12 months of treatment (*p* < 0.001) (Table 2). This improvement proved to be stable and durable over time, with a median score reaching 9.0 (IQR 5.0–11.0) at the 24-month follow-up (*p* < 0.001 vs. baseline). The comparison between the 12- and 24-month timepoints showed no statistically significant differences (*p* = 0.75), confirming the maintenance of the clinical benefit in the long term.

OCS usage dropped to 0 mg/day by month 12 and was maintained through month 24 (*p* < 0.001) (Table 2). Notably, asthma metrics (ACT, FEV_1_) showed no significant difference between 12 and 24 months, indicating maximum benefit is reached early. The totality of patients who achieved symptomatic asthma control at 24 months also demonstrated FEV_1_ stability or improvement (*p* = 0.017 vs. baseline), thus fulfilling the complete SANI remission criteria. Assessment of lung volumes via body plethysmography revealed a significant reduction in residual volume (RV) at 12 months, decreasing from a median of 1.86 L to 1.79 L (*p* = 0.022). Interestingly, total lung capacity (TLC) showed a significant increase by month 24 (from 5.92 L to 6.19 L, *p* = 0.042), further supporting the overall improvement in pulmonary volumes. In contrast, nasal polyps progressively shrank through follow-up (Figure 2), with NPS showing a statistically significant reduction between month 12 and month 24 (*p* < 0.05).

### 3.3. Safety

No serious adverse events were recorded, and there were no discontinuations due to adverse events. Transient asymptomatic blood hypereosinophilia (≥1500 cells/µL) was observed in 3 patients (11%) at 12 months and 1 patient (6%) at 24 months, with no high-grade hypereosinophilia (>3000 cells/µL) (Table 3).

### 3.4. Dual Remission and Complete Recovery

Asthma remission was achieved rapidly and maintained at high rates: 85.7% (24/28) of patients achieved remission at 12 months, rising to 100% (25/25 of the remaining patients) by 24 months in the observed population. In contrast, CRSwNP remission showed a higher initial response rate than previously estimated, followed by continued improvement. At 12 months, 57% (16/28) of patients met the criteria for CRSwNP remission. This rate increased substantially to 88% (22/25) at 24 months, confirming that tissue remodeling continues well into the second year of therapy.

**Figure 3:**
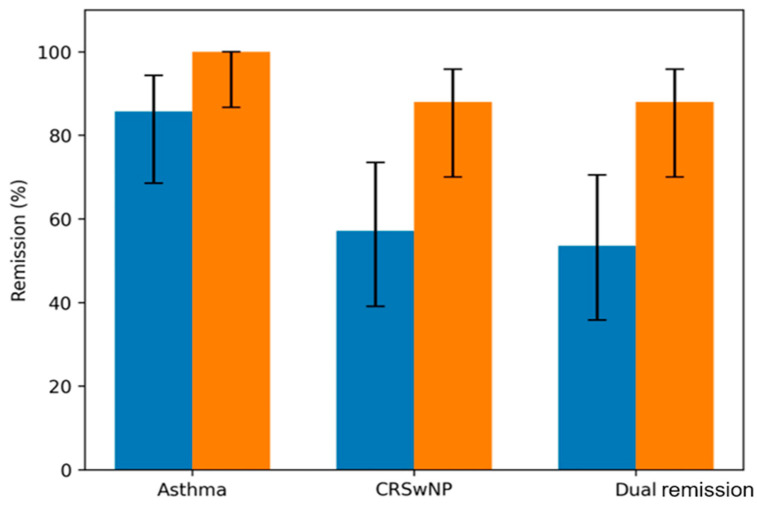
Evolution of single and dual remission rates. Bar chart displaying the percentage of patients achieving remission criteria at 12 and 24 months. Error bars represent 95% confidence intervals (Wilson method). The blue bars show the 12-month trend and the orange bars the 24-month trend. Abbreviations: CRSwNP: Chronic Rhinosinusitis with nasal Polyps.

Consequently, the rate of “Dual Remission” (simultaneous achievement of both asthma and CRSwNP remission) rose from 54% (15/28) at year 1 to 88% (22/25) at year 2. Notably, 70% of patients who had not achieved CRSwNP remission at year 1 (due to residual polyp scores >1) successfully achieved it by year 2 (Table 4 and Figure 3). When adding normosmia to the remission criteria, the rate of “Complete Recovery” was 32% (8/25), revealing that 56% of patients achieved clinical silence but remained hyposmic.

## 4. Discussion

This study confirms that dupilumab is a potentially transformative therapy for patients with severe T2 respiratory comorbidities, capable of inducing a state of “clinical silence”. Our main findings showed that dual remission increased substantially from 53.6% at 12 months to 88.0% at 24 months (Figure 3). The most critical insight is the temporal dissociation between asthma and CRSwNP remission. While 100% of patients achieved asthma remission within the observed period, endoscopic normalization required a longer duration (Table 4). This “lag” suggests fundamental differences in tissue responsiveness: bronchial smooth muscle dysfunction resolves rapidly, whereas nasal polyps represent substantial tissue remodeling that requires a longer duration to regress. Clinical control often aligns with polyp regression in the majority of patients (57%) by year 1. However, the significant leap to 88% remission at year 2 validates the “catch-up” phenomenon.

Our cohort achieved substantially higher asthma remission rates (100% at 24 months) compared to the 37–43% reported in the QUEST and TRAVERSE trials [3,4]. This result must be interpreted in light of the specific composition of our population. As detailed in Table 1, 71% of our cohort suffered from mild-to-moderate asthma, while only 28.6% had severe asthma. This prevalence of mild-to-moderate asthma phenotypes, driven primarily by the severe CRSwNP indication, likely contributed to the high ceiling of asthma remission observed.

Achieving “remission” (no exacerbations, stable lung function) is inherently more attainable in mild-to-moderate disease once the upper airway trigger is controlled, compared to refractory severe asthma [15]. However, the fact that remission was achieved in the entire cohort, including the 8 patients with severe disease, underscores the biologic’s efficacy across the severity spectrum.

Furthermore, viewing our results through the lens of the VESTIGE trial reinforces the concept of “united airway remodeling.” In VESTIGE, clinical improvements in lung function were mechanistically linked to the reduction in airway mucus volume and inflammation (FeNO) [16]. Similarly, in our study, the eventual achievement of dual remission likely relies on the same IL-4/13 blockade reducing fluid extravasation and fibrin deposition in both the lungs and sinuses. The contrast we observed—rapid asthma control versus delayed polyp regression—mirrors the kinetic difference often seen between functional biomarkers and structural imaging endpoints. This reinforces the hypothesis that while “clinical control” (symptoms) can be achieved early, “structural remission” (tissue normalization) is a prolonged event that accrues well beyond the first year of therapy.

Our findings resonate with the real-life data presented by De Corso et al. in their consecutive multicentric analyses. In their 2022 study, De Corso et al. observed that while patient-reported outcomes (SNOT-22) improve dramatically and rapidly, the reduction in the polypoid burden (NPS) follows a more gradual slope [17]. Furthermore, in the large-scale DUPIREAL study, De Corso et al. reported that while 96.9% of patients were classified as moderate/excellent responders at 12 months, the achievement of full clinical remission requires sustained therapy [18]. Both our single-center data and their multicentric findings convergently support the conclusion that patient-reported “response” often precedes objective “remission,” and that dupilumab requires prolonged administration to fully reverse structural remodeling.

This dissociation is also evident in olfactory recovery. In our cohort, we observed a progressive restoration of smell, with median SS-I scores tripling from 3 at baseline to 9 at 24 months (*p* < 0.001). However, distinct from the structural improvement (NPS) that continued significantly into the second year, olfactory function stabilized after the first 12 months (*p* = 0.75 for T12 vs. T24), suggesting that functional recovery may plateau earlier than anatomical normalization. This objective improvement aligns with the dramatic reduction in SNOT-22 scores, yet it highlights a crucial area of divergence in the literature regarding remission definitions. Our observed dual remission rate of 88% at 24 months is notably higher than the 31% reported by Tajiri et al. [19].

This difference likely reflects our definition of remission, which accepts “clinical silence” and minor mucosal thickening, whereas other studies may demand absolute olfactory restitution and total polyp disappearance [20]. While our study demonstrates that dupilumab significantly improves olfactory performance (SS-I score 9.00 at 24 months [*p* < 0.001 vs. baseline]), requiring “complete” restitution—which may be limited by long-standing neuroepithelial damage—sets a much higher bar than the EPOS/EUFOREA criteria used in our analysis [13]. Thus, while smell improves substantially, the definition of “remission” heavily influences reported success rates in real-world studies. In this regard, while 88% of our cohort achieved Dual Remission at 24 months, indicating effective control of type 2 inflammation in both the upper and lower airways, the restoration of olfactory function followed a distinct and less complete trajectory. When we applied a stricter composite definition requiring Dual Remission plus normosmia (SS-I score ≥ 12), the success rate dropped to 32% (8/25 patients), overlapping with the data from the study by Tajiri et al. [19]. This observation reveals a significant ‘clinical–functional gap’ affecting approximately 56% of our patients (14/25), who achieved clinical silence (no asthma exacerbations, OCS independence, and reduced polyp burden) but remained hyposmic. This discrepancy strongly supports the hypothesis that long-standing CRSwNP may cause irreversible neuroepithelial damage or remodeling that persists even after the inflammatory burden is resolved. Consequently, while Dupilumab proves highly effective at inducing clinical remission, ‘complete’ recovery including full olfactory restitution may require longer treatment duration or may not be achievable for all patients with severe, chronic disease.

Our data also integrates the recently published “Two-Year Turning Point” study, the largest real-life investigation to date involving 926 patients up to 1 year [20]. These authors demonstrated that patients who had not yet achieved optimal control targets at 12 months showed continued significant improvement during the second year of therapy. This confirms our observation of a “catch-up” phenomenon on a much larger scale, validating the concept that 12 months is often an insufficient horizon to judge the full potential of biologic therapy in tissue remodeling. Furthermore, they found that tapering the dosage to every 4 weeks was feasible in nearly 19% of patients without loss of control, suggesting that once the “turning point” of deep remission is reached, the therapeutic burden can be reduced. The novelty of our findings lies in the “catch-up” phenomenon: 70% who had not achieved CRSwNP remission at 1 year achieved it by year 2 (6 out of 9 evaluated non-responders) (Figure 2). This argues strongly against switching therapies prematurely if a patient has excellent asthma control but “sub-optimal” polyp reduction at 12 months.

These real-world observations regarding the kinetics of remission offer a complementary long-term perspective to the recently published EVEREST trial [21]. While this head-to-head trial validated the early superiority of dupilumab over omalizumab at 24 weeks, our 24-month data suggest that the full extent of structural remodeling—particularly in the upper airways—accrues well beyond the timeframe of substantial clinical trials. Furthermore, the high consistency of dual remission observed in our cohort contrasts with recent transcriptomic findings by Estravís et al., who identified specific biomarkers (e.g., RGS1) as necessary predictors for a “dual super-response” to mepolizumab [22]. This distinction reinforces the hypothesis that the broad upstream blockade of IL-4 and IL-13 may induce “clinical silence” across a wider phenotypic spectrum compared to the more biomarker-dependent efficacy observed with distal cytokine inhibition.

Our observed dual remission rate of 88% at 24 months is notably higher than the 31% reported by Tajiri et al. [19]. This significant discrepancy is primarily attributable to the different stringencies of the remission definitions applied in the two studies. While our analysis utilized the EPOS/EUFOREA criteria for CRSwNP remission [13], which defines success as a state of ‘controlled’ low-grade disease (NPS ≤ 1 and SNOT-22 < 40), Tajiri et al. employed a much stricter composite definition requiring absolute endoscopic silence and, crucially, the complete restitution of olfactory function. To further investigate this divergence, we applied a stricter composite definition to our cohort, requiring Dual Remission plus objective normosmia (SS-I score ≥ 12). Under these more rigorous criteria, our success rate dropped to 32% (8/25 patients), a result that closely aligns with the 31% reported by Tajiri et al. This comparison reveals a significant ‘clinical–functional gap’: while the majority of patients achieve structural and symptomatic control (clinical remission), full sensory recovery remains more elusive. This supports the hypothesis that long-standing CRSwNP may cause irreversible neuroepithelial damage that persists even after the inflammatory and polypoid burden is resolved. Consequently, the reported success of biologic therapy in real-world settings is heavily influenced by whether the therapeutic goal is defined as ‘clinical silence’ or ‘complete biological and functional clearance’.

A noteworthy finding of our study is the significant reduction in RV observed at 12 months, which decreased from a median of 1.86 L to 1.79 L (*p* = 0.022). This physiological improvement provides clinical evidence of a reduction in small airway air trapping, a hallmark of Type 2-driven airway remodeling. While traditional metrics like FEV_1_ reflect large airway patency, the evolution of RV suggests that IL-4/13 blockade may effectively target distal airway inflammation and mucus plugging. This is consistent with recent findings from the VESTIGE trial, which utilized functional respiratory imaging to demonstrate Dupilumab’s ability to reduce inflammatory indicators in the lung periphery [16]. Interestingly, while RV improved early, we observed a significant increase in TLC by month 24, potentially reflecting a long-term recovery of overall lung compliance and volumes as the ‘united airway’ inflammatory burden is progressively cleared [23].

Regarding biological biomarkers, our data on FeNO showed no statistically significant variations over the 24-month period. This finding contrasts with several previous reports and clinical trials, such as QUEST and VESTIGE [3,16], which demonstrated a rapid and sustained decrease in FeNO levels following dupilumab treatment. However, it is important to note that our cohort presented relatively low median FeNO levels at baseline (23.0 ppb). This baseline characteristic may have limited the measurable impact of IL-4/13 blockade on this specific biomarker, a phenomenon also observed in other real-life studies where patients with lower baseline FeNO exhibit less pronounced biological shifts compared to those with high-T2 endotypes. Furthermore, the lack of significant FeNO reduction in our study does not preclude clinical efficacy, as evidenced by the 100% asthma remission rate. This suggests that in some ‘united airway’ phenotypes, clinical and functional stability can be achieved even in the absence of dramatic changes in exhaled inflammatory markers [24].

Beyond the clinical benefits, the achievement of dual remission has significant pharmacoeconomic implications. The high acquisition cost of monoclonal antibodies is a frequent subject of debate; however, the concept of “cross-coverage”—treating two severe, resource-intensive comorbidities with a single agent—substantially improves the therapy’s value proposition [25]. Jommi et al. analyzed this “crowding-out” effect from the Italian National Health Service (NHS) perspective, estimating that the use of dupilumab for cross-coverage (treating CRSwNP in patients with severe asthma and vice versa) generates significant savings by displacing the costs of alternative treatments, surgeries, and comorbidity management. Their model estimated total potential annual savings ranging from €0.95 to €8.36 million, depending on market penetration [25].

These findings are supported by a specific cost-utility analysis for Italy conducted by De Corso et al., which demonstrated that dupilumab as an add-on treatment for severe uncontrolled CRSwNP is cost-effective. They reported an incremental cost–utility ratio (ICUR) of €21,817 per quality-adjusted life year (QALY) gained, a figure well below the commonly accepted willingness-to-pay thresholds in the Italian healthcare system (€25,000–€40,000/QALY) [26]. International data further contextualizes this: in South Korea, Oh et al. found that add-on dupilumab was cost-effective for severe asthma (ICER 20,325/QALY), driven largely by the reduction in severe exacerbations and improvements in quality of life [27]. However, cost-effectiveness is sensitive to local pricing and comparators; a Canadian study by Yong et al. noted that while dupilumab demonstrated superior efficacy in SNOT-22 improvement compared to omalizumab and mepolizumab, its relative cost-effectiveness was heavily dependent on drug acquisition costs and dosing frequency [28]. Therefore, the “dual remission” observed in our real-world cohort—where 100% of asthma patients and 81.3% of CRSwNP patients achieved remission at 24 months—likely represents the scenario of maximum economic efficiency, where the single biologic cost is offset by the simultaneous cessation of resource utilization in both the upper and lower airways.

## 5. Study Limitations

Despite the clinical relevance of our findings, this study has some limitations that warrant consideration. First, the retrospective design may have introduced inherent biases in data collection and patient selection. Second, the sample size is relatively small ($n = 28$), which may limit the generalizability of our results to broader populations and reduce the statistical power for secondary endpoints. Third, the high rate of asthma remission should be interpreted with caution, as it likely reflects a ‘united airway’ phenotype where upper airway disease was the primary driver of symptoms, rather than a cohort of purely refractory severe asthma. Finally, as a single-center study, our findings reflect the clinical practice and patient management of a specific multidisciplinary unit.

## 6. Conclusions

In conclusion, our 24-month real-world data suggest that dupilumab is a highly effective therapeutic option for achieving simultaneous clinical remission in patients with comorbid asthma and CRSwNP. The ‘catch-up’ phenomenon observed in our cohort—where 70% of those who had not achieved nasal remission at one year did so by year two—indicates that clinical silence is a realistic target that often requires a 24-month horizon to manifest fully. However, the persistence of a ‘clinical–functional gap,’ where 56% of patients remain hyposmic despite structural polyp regression, highlights the potential for irreversible neuroepithelial damage in long-standing disease. Therefore, we recommend the earlier inclusion of biologic therapy in the treatment algorithm, before such permanent structural and sensory dissociation occurs, to shift the therapeutic goal from symptomatic control to complete functional recovery.

## Figures and Tables

**Table 1:** Baseline demographic and clinical characteristics of the 28 patients included in this study.

Variable	Value (N = 28)
Demographics	
Age, years	53.50 (48.75–61.25)
Sex, n (%)	Male: 17 (61%)/Female: 11 (39%)
BMI, kg/m^2^	24.75 (22.75–28.00)
Disease history & severity	
Asthma severity, n (%)	
Severe asthma	8 (29%)
Mild-Moderate asthma	20 (71%)
Asthma duration, years	12.00 (2.00–21.75)
CRSwNP duration, years	12.50 (8.00–20.50)
Comorbidities (NSAID-ERD), n (%)	12 (42.9%)
Baseline clinical metrics	
Blood eosinophils, cells/µL	525.00 (400.00–652.50)
Total IgE, IU/mL	164.50 (101.75–324.25)
FEV_1_, L	3.12 (2.56–3.61)
ACT score	19.00 (16.50–24.00)
SNOT-22 score	67.00 (54.50–80.00)
Nasal Polyp Score (NPS) (0–8)	6.00 (6.00–6.00)
Daily OCS, mg/day	1.40 (0.00–2.90)

Data are presented as Median (IQR 25th–75th percentile) for continuous variables and n (%) for categorical variables. N = 28 unless otherwise specified. Abbreviations: BMI, Body Mass Index; CRSwNP, Chronic Rhinosinusitis with Nasal Polyps; NSAID-ERD, Non-Steroidal Anti-Inflammatory Drug-Exacerbated Respiratory Disease; IgE, Immunoglobulin EFEV_1_, Forced Expiratory Volume in 1 s; ACT, Asthma Control Test; SNOT-22, Sino-Nasal Outcome Test-22; NPS, Nasal Polyp Score; OCSs, Oral Corticosteroids.

**Table 2:** Evolution of clinical, functional, and biological parameters during Dupilumab treatment.

Parameter	Baseline	12 Months	24 Months	*p*-Value (12 m vs. Baseline)	*p*-Value (24 m vs. Baseline)
Clinical control					
ACT score	19 (16.5–24)	25 (25–25)	25 (25–25)	<0.001	0.007
SNOT-22 score	67 (54.5–80)	10 (5–15)	5 (3–13.25)	<0.001	<0.001
NPS (0–8)	6 (6–6)	0.5 (0–2)	0 (0–0)	<0.001	<0.001
Sniffin’ Stick score	3 (3–3)	7 (5.25–9.75)	9 (5–11)	<0.001	<0.001
OCS (mg/day)	1.4 (0.0–2.9)	0.0 (0.0–0.0)	0.0 (0.0–0.0)	<0.001	<0.001
Lung function					
FEV_1_ (L)	3.12 (2.56–3.61)	3.50 (3.05–3.83)	3.50 (3.11–3.91)	0.064	0.018
FEV_1_ (% predicted)	94.00 (84.00–111.8)	102.50 (90.25–111.3)	109.00 (101.5–117.8)	0.127	0.017
FVC (L)	4.53 (3.87–5.21)	4.42 (4.37–5.99)	4.57 (4.13–5.40)	0.433	0.330
FVC (% predicted)	112.00 (102.5–120.5)	116.50 (109.3–121.5)	117.50 (108.5–123.0)	0.426	0.469
FEF25–75 (L/s)	1.76 (1.27–2.88)	2.34 (1.45–2.83)	2.74 (1.74–2.95)	0.231	0.635
FEF25–75 (% pred)	54.00 (41.00–92.50)	70.00 (51.50–88.75)	88.00 (66.50–114.5)	0.071	0.569
Lung volumes					
RV (L)	1.86 (1.35–2.56)	1.79 (1.47–2.07)	1.87 (1.46–2.17)	0.022	1.000
TLC (L)	5.92 (4.84–6.73)	6.15 (5.77–6.93)	6.19 (5.85–7.38)	0.241	0.042
Biomarkers					
Blood eosinophils (/µL)	525 (400–653)	535 (405–1195)	660 (140–1033)	0.053	0.229
FeNO (ppb)	23.00 (22.00–90.00)	37.00 (28.00–48.00)	25.00 (19.00–30.00)	0.500	1.000

Data are presented as Median (IQR 25th–75th). *p*-values calculated using Wilcoxon signed-rank test. Abbreviations: ACT, Asthma Control Test; SNOT-22, Sino-Nasal Outcome Test-22; NPS, Nasal Polyp Score; OCS, Oral Corticosteroids; FEV_1_, Forced Expiratory Volume in 1s; FVC, Forced Vital Capacity; FEF25–75, Forced Expiratory Flow at 25–75% of FVC; RV, Residual Volume; TLC, Total Lung Capacity; FeNO, Fractional exhaled Nitric Oxide.

**Table 3:** Safety profile and blood eosinophil evolution.

Parameter	Baseline (N = 28)	12 Months (N = 28)	24 Months (N = 25)
Hypereosinophilia			
Eosinophils ≥ 1500 cells/µL, n (%)	0 (0%)	3 (11%)	1 (6%)
Eosinophils > 3000 cells/µL, n (%)	0 (0%)	0 (0%)	0 (0%)
Adverse events, n (%)			
Serious infections *	0 (0%)	0 (0%)	0 (0%)
Discontinuation due to AE	-	0 (0%)	0 (0%)

Data are presented as number (percentage) of patients. Eosinophil counts refer to absolute blood eosinophil levels. * No severe infections or injection site reactions requiring treatment discontinuation were reported during the 24-month observation period.

**Table 4:** Comprehensive clinical & olfactory outcomes.

Outcome Measure	Baseline (N = 28)	T12 (12 m) (N = 28)	T24 (24 m) (N = 25)
Asthma remission (SANI criteria)	0%	85.7%	100%
CRSwNP Remission (EPOS/EUFOREA)	0%	57.1%	88.0%
Dual Remission (Asthma + CRSwNP)	0%	53.6%	88.0%
Complete recovery (Dual Rem. + normosmia)	0%	-	32.0%

Values expressed as n/N (%). N varies due to data availability at each timepoint. Abbreviations: SANI: Severe Asthma Network Italy; EPOS: European Position Paper on Rhinosinusitis and Nasal Polyps; EUFOREA: European Forum for Research and Education in Allergy and Airway Diseases; CRSwNP: Chronic Rhinosinusitis with Nasal Polyps.

## Data Availability

The data supporting the findings of this study are available from the corresponding author upon reasonable request.

## Abbreviations

ACT	Asthma Control Test
OCS	Oral Corticosteroids
SNOT-22	Sino-Nasal Outcome Test-22
NPS	Nasal Polyp Score
BMI	Body Mass Index
CRSwNP	Chronic Rhinosinusitis with Nasal Polyps
FEF25–75	Forced Expiratory Flow at 25–75% of FVC
FeNO	Fractional exhaled Nitric Oxide
FEV1	Forced Expiratory Volume in 1 s
FVC	Forced Vital Capacity
GERD	Gastroesophageal Reflux Disease
ICS	Inhaled Corticosteroids
ICUR	Incremental Cost-Utility Ratio
IgE	Immunoglobulin E
IL-4	Interleukin-4
IL-5	Interleukin-5
IL-13	Interleukin-13
IQR	Interquartile Ranges
LABA	Long-Acting Beta-Agonists
LAMA	Long-Acting Muscarinic Antagonists
NHS	National Health Service
NSAID-ERD	Non-Steroidal Anti-Inflammatory Drug-Exacerbated Respiratory Disease
QALY	Quality-Adjusted Life Year
RV	Residual Volume
SANI	Severe Asthma Network Italy
SS-I	Sniffin’ Sticks Identification test
T2	Type 2 (inflammation)
TLC	Total Lung Capacity
